# Coronary Artery-Specific Changes in Patients with Chronic Kidney Disease

**DOI:** 10.3390/cells15090765

**Published:** 2026-04-24

**Authors:** Julia Hanke, Katarzyna Romejko, Stanisław Niemczyk

**Affiliations:** Department of Internal Diseases, Nephrology and Dialysis, Military Institute of Medicine—National Research Institute, 128 Szaserów Street, 04-141 Warsaw, Poland; kromejko@wim.mil.pl (K.R.); sniemczyk@wim.mil.pl (S.N.)

**Keywords:** chronic kidney disease, coronary artery disease, atherosclerosis, coronary artery calcification, endothelial dysfunction

## Abstract

Cardiovascular disease represents the primary cause of morbidity and mortality among patients with chronic kidney disease (CKD). Emerging evidence suggests that coronary artery pathology in CKD diverges from the traditional atherosclerotic phenotype seen in individuals with maintained renal function. This review delineates coronary artery-specific alterations in CKD, focusing on mechanisms that expedite atherogenesis, characteristics of plaques, calcific remodeling, and dysfunction of the coronary microvasculature. CKD fosters a pro-inflammatory, pro-oxidative, and pro-calcific environment, which results in endothelial damage and vascular calcification remodeling. Furthermore, coronary plaques in CKD are more likely to exhibit larger lipid-rich necrotic cores, heightened inflammatory cell infiltration, a significant calcific burden, and vulnerability indicators such as cholesterol crystals and microdisruptions. Impaired coronary microvascular function is also prevalent and may account for ischemia with non-obstructive coronary arteries. In summary, CKD is linked to a rapid, calcification- and inflammation-driven form of coronary disease characterized by both macrovascular plaque remodeling and microvascular dysfunction. This underscores the necessity of early identification and prevention of cardiovascular risk, targeting CKD-specific mechanisms in conjunction with conventional risk factors.

## 1. Introduction

Cardiovascular complications such as atherosclerosis, coronary and peripheral artery disease, hypertension and heart failure occur in the early stages of chronic kidney disease (CKD) and develop as renal function declines, being the main cause of morbidity and mortality in this group. Due to the increasing prevalence of CKD, which currently affects approximately 10% of the global population and continues to rise, potentially becoming the fifth leading cause of death by 2040, understanding the specificity of cardiovascular complications in this population is of essential significance. Early diagnosis of cardiovascular disorders in CKD patients is crucial, as it enables the implementation of appropriate treatment and preventive interventions, thereby slowing disease progression and prolonging life expectancy.

Traditional risk factors for atherosclerosis are highly prevalent in CKD patients and substantially contribute to the excess burden of coronary artery disease (CAD). Patients with CKD exhibit a significant accumulation of traditional cardiovascular risk factors such as advanced age, arterial hypertension, diabetes mellitus, dyslipidemia and physical inactivity. The prevalence and clustering of these factors intensify as glomerular filtration rate decreases and play a major, often additive, role in the risk of CAD across all stages of CKD [1,2]. However, along with traditional atherosclerotic risk factors, there are numerous pathological states specific to CKD which predispose to the early occurrence and the accelerated progression of atherosclerosis in this group, like increased inflammation, calcium-phosphate metabolic disturbances, oxidative stress (OS), endothelial dysfunction, elevated advanced glycation end products (AGEs) and uremic toxins, as well as lipoprotein modifications.

**Figure 1:**
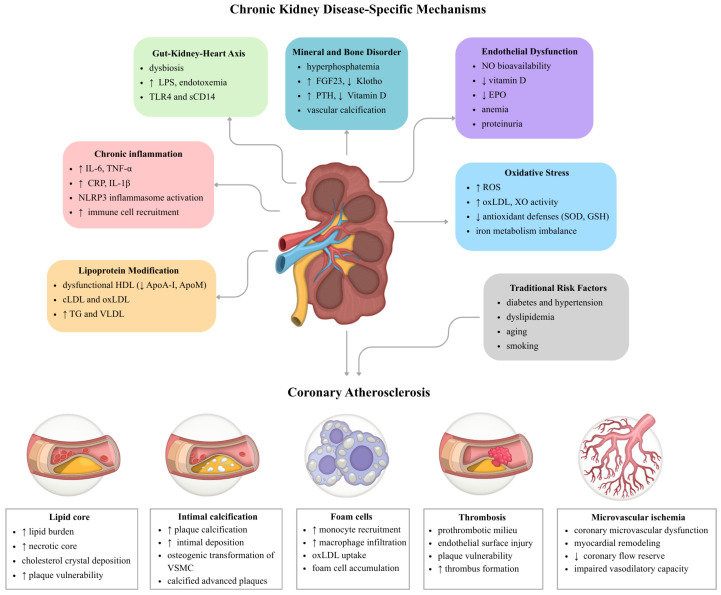
CKD-specific mechanisms of coronary atherosclerosis. ApoA-I, apolipoprotein A-I; ApoM, apolipoprotein M; cLDL, carbamylated low-density lipoprotein; CKD, chronic kidney disease; CRP, C-reactive protein; EPO, erythropoietin; FGF23, fibroblast growth factor 23; GSH, glutathione; HDL, high-density lipoprotein; IL-1β, interleukin 1 beta; IL-6, interleukin 6; LPS, lipopolysaccharide; NLRP3, NLR family pyrin domain containing 3; NO, nitric oxide; oxLDL, oxidized low-density lipoprotein; PTH, parathyroid hormone; ROS, reactive oxygen species; sCD14, soluble cluster of differentiation 14; SOD, superoxide dismutase; TG, triglycerides; TLR4, Toll-like receptor 4; TNF-α, tumor necrosis factor alpha; VLDL, very-low-density lipoprotein; VSMC, vascular smooth muscle cell; XO, xanthine oxidase;. ↑, increase or upregulation; ↓, decrease or downregulation.

According to recent research, atherosclerotic plaques in patients with CKD are more complex and unstable than those in individuals with preserved renal function [3]. CKD-related plaques have larger lipid-rich necrotic cores, more extensive calcium deposits, and increased inflammatory cell infiltration, which results in greater plaque vulnerability. Imaging studies indicate that patients with CKD exhibit a higher prevalence of cholesterol crystal formation and calcification within coronary plaques, as well as an increased incidence of small plaque disruptions, compared to the non-CKD population. Severe calcification, persistent inflammation, and soft lipid components are the characteristic plaque features of CKD that support an accelerated type of coronary atherosclerosis in this group. Figure 1 illustrates the mechanisms responsible for CKD-associated alterations in the coronary arteries and summarizes their key features.

CKD is a heterogeneous condition that does not progress uniformly across all patients. Its clinical course and vascular consequences are shaped by multiple factors, including genetic background, comorbidities, and the underlying cause of kidney dysfunction. Therefore, the aim of this review is to outline the principal mechanisms associated with CKD that may contribute to coronary artery alterations.

## 2. Factors Which Significantly Contribute to the Development of Atherosclerosis in CKD

### 2.1. Chronic Inflammatory State

CKD is associated with a persistent low-grade inflammatory state that contributes to accelerated atherosclerosis and to the high cardiovascular burden observed in this population [4]. Impaired renal clearance of uremic toxins promotes activation of innate immune processes, with increased circulating concentrations of pro-inflammatory mediators, including interleukin-6 (IL-6) and tumor necrosis factor-alpha (TNF-α) [4,5]. In patients with advanced CKD and in those receiving dialysis, higher concentrations of IL-6 and TNF-α were repeatedly reported, supporting the concept that uremia is accompanied by systemic inflammatory activation [5,6,7]. TNF-α triggers inflammatory cascades and stress-response pathways, including caspases, nuclear factor kappa B (NF-κB) and mitogen-activated protein kinase pathways, which potentiate vascular inflammation and tissue damage [4]. In experimental settings, TNF-α and IL-6 were also implicated in osteogenic transdifferentiation and calcification of vascular smooth muscle cells, partly through the activator protein 1/cellular Fos proto-oncogene protein-dependent signaling [7].

Experimental evidence further supports a proatherogenic role of IL-6, particularly during early lesion development [8,9]. In a murine model, administration of recombinant IL-6 exacerbated early atherosclerotic lesion formation and amplified the proinflammatory cytokine milieu, including interleukin-1 beta (IL-1β) and TNF-α [9]. Subsequent mechanistic studies showed that IL-6 promotes endothelial dysfunction by impairing endothelial nitric oxide synthase/nitric oxide (eNOS/NO) signaling and increasing OS [10]. Moreover, IL-6 trans-signaling appears to be particularly important for vascular inflammation and atherosclerotic plaque progression in experimental atherosclerosis [8]. Local vascular induction of IL-6 was also linked to Janus kinase/signal transducer and activator of transcription 3 pathway activation during early atherogenesis [11]. Also, in dialysis-dependent CKD, activation of the NLR family pyrin domain containing 3 inflammasome was demonstrated in peripheral blood mononuclear cells, together with increased activation of caspase-1 and enhanced maturation of IL-1β and interleukin 18 (IL-18) [12]. These findings support the concept that IL-1β-mediated inflammatory signaling is active in uremia, and clinical evidence from anti-inflammatory intervention studies further supports the relevance of this pathway in contributing to residual cardiovascular risk [12,13]. Although some knockout studies suggest that lifelong complete IL-6 deficiency may have paradoxical effects on plaque development, the overall experimental evidence supports a context-dependent but biologically relevant pro-atherogenic role of IL-6 [14].

Additionally, TNF-α and IL-6 upregulate adhesion molecules and chemokines, facilitating leukocyte recruitment and binding to the vessel wall, thereby promoting atherosclerotic plaque development [5].

IL-6 is a major inducer of hepatic C-reactive protein (CRP) synthesis, and circulating CRP therefore reflects activation of the upstream IL-1β/IL-6 inflammatory axis [15]. However, increased CRP serum concentrations in CKD are not only due to higher IL-6 levels but also to reduced clearance, leading to CRP accumulation. Approximately 25% of CKD patients have CRP concentrations above 5 mg/L, and around 50–70% of patients undergoing dialysis have serum CRP levels exceeding typical risk thresholds of 5–10 mg/L [16,17,18]. This indicates that inflammatory processes activation begins at early stages of renal failure and intensifies as kidney function declines [19]. Importantly, CRP should be interpreted primarily as a downstream biomarker of systemic inflammation rather than as definitive evidence of a direct causal mediator of plaque formation [15,20]. Consistent with this interpretation, local sampling of ruptured coronary plaques demonstrated increased IL-6 but not CRP, suggesting that CRP predominantly reflects systemic inflammatory activation rather than local plaque synthesis in that setting [20]. Original studies in predialysis CKD confirmed inverse associations between kidney function and circulating CRP and IL-6, indicating that inflammation is already detectable before the initiation of dialysis and intensifies as renal dysfunction progresses [18,19]. Numerous studies found an association between increased inflammatory markers, such as CRP, IL-6, and TNF-α, and higher cardiovascular risk in CKD. A meta-analysis of Li, which included 32 studies, found that CKD patients with increased serum CRP had an approximately 20% higher risk of all-cause and cardiovascular mortality [21]. Similarly, a pooled analysis of 25 prospective studies showed that each standard deviation increase in serum IL-6 and TNF-α concentrations was associated with a higher risk of fatal coronary events of more than 25% and 17%, respectively [22]. High-sensitivity CRP (hsCRP) may thus serve as a clinical biomarker of inflammation-mediated atherosclerotic risk, owing to its strong epidemiological associations with atherosclerosis and the widespread availability of its assay. However, IL-6 may represent a more accurate marker of activation of the IL-1β/IL-6 inflammatory pathway underlying this, ‘residual inflammatory risk’, as CRP reflects only the terminal downstream product of that cascade [15].

Importantly, the relationship between circulating inflammatory markers and cardiovascular outcomes may be influenced by reverse causation. Vascular injury and atherosclerotic burden can themselves drive systemic inflammation, particularly in CKD, a condition characterized by a persistent inflammatory baseline. Thus, elevated levels of CRP, IL-6, and TNF-α may reflect both cause and consequence of vascular disease. Given that most evidence is observational, the directionality of these associations cannot be definitively established, and caution is warranted when inferring causality [23,24,25].

### 2.2. Calcium-Phosphate Metabolic Disturbances

The development of chronic kidney disease–mineral and bone disorder (CKD-MBD), encompassing hyperphosphatemia, secondary hyperparathyroidism, vitamin D deficiency, elevated circulating fibroblast growth factor 23 (FGF23), and Klotho protein deficiency represents a common and clinically relevant consequence of CKD progression. These disturbances emerge early in the course of renal dysfunction and contribute not only to skeletal abnormalities but also to cardiovascular changes [26,27,28].

Among these alterations, phosphate retention appears to play a central pathophysiological role. Increased serum phosphate concentrations were associated with a higher risk of cardiovascular events in CKD patients [29,30]. Hyperphosphatemia, primarily resulting from impaired renal phosphate excretion significantly contributes to vascular calcification, and therefore cardiovascular risk [29]. At the cellular level, extracellular phosphate is transported into vascular smooth muscle cells (VSMC) through sodium-dependent phosphate transporters, where it promotes their osteogenic transdifferentiation into osteoblast-like cells and enhances hydroxyapatite deposition within the arterial wall, thereby accelerating vascular calcification [31,32]. In addition to its pro-calcific effects, phosphate excess adversely affects endothelial function. Elevated phosphate exposure was shown to impair endothelium-dependent vasodilation, increase reactive oxygen species (ROS) generation, reduce eNOS activity, and diminish nitric oxide bioavailability, thereby predisposing to vasoconstriction and vascular dysfunction [33,34,35]. In patients undergoing either hemodialysis (HD) or peritoneal dialysis (PD), advanced phosphate overload was also associated with more severe coronary calcification. This profound mineral imbalance may also have direct interventional cardiology implications, as extensive vascular calcification in dialysis patients may contribute to calcified nodule formation within stented coronary segments, a histopathologically documented mechanism of early in-stent restenosis [36].

The FGF23/Klotho axis constitutes another major pathogenic link between CKD-MBD and cardiovascular injury [37,38]. FGF23 increases early during CKD progression, often before overt hyperphosphatemia develops, as a compensatory response aimed at maintaining phosphate balance by enhancing renal phosphate excretion. However, this adaptive mechanism simultaneously suppresses renal 1,25-dihydroxyvitamin D synthesis and may thereby contribute to calcitriol deficiency [39]. Beyond its phosphaturic effects, FGF23 was implicated in the pathogenesis of cardiovascular dysfunction. Both experimental and clinical evidence suggest that elevated FGF23 may contribute to endothelial dysfunction and thereby promote adverse vascular remodeling and atherosclerosis-related processes [40,41,42]. FGF23 was shown to stimulate hepatic expression of CRP and IL-6, thereby exacerbating the systemic inflammatory response in CKD [43]. Elevated serum FGF23 concentrations were associated with the severity of arterial calcification, including coronary artery calcification measured by the Agatston score, as well as with intima-media complex thickening and increased arterial stiffness [40,44,45]. Notably, a recent meta-analysis including 27,459 participants demonstrated that FGF23 and Klotho were significantly associated with arterial calcification, arterial thickness, and arterial stiffness, further strengthening the concept that this signaling axis contributes to vascular remodeling [40]. Additionally, experimental studies suggested that FGF23 may directly enhance vascular calcification by promoting the osteogenic differentiation of VSMC, potentially through the extracellular signal-regulated kinase 1/2 (ERK1/2) pathway, particularly under high-phosphate conditions [32].

In contrast, Klotho, which is predominantly synthesized in the kidneys and functions as a co-receptor for FGF23, appears to exert vasculoprotective effects [46,47]. Klotho promotes phosphate excretion and suppresses vascular calcification through multiple mechanisms, including favorable modulation of calcium-phosphate homeostasis and direct anti-osteogenic effect on vascular cells [46]. The reduction in renal Klotho expression observed in CKD, together with lower circulating concentrations of soluble Klotho, was associated with vascular calcification, arterial stiffness, and worse cardiovascular outcomes [46,48,49]. Experimental observations additionally demonstrated that Klotho expression in aortic smooth muscle cells declines progressively with CKD severity, being significantly reduced in CKD stage 3, further suppressed in CKD stage 4/5, and nearly completely inhibited in hemodialysis patients [50]. Moreover, reduced Klotho signaling in CKD favors the osteogenic conversion of VSMC, thereby contributing to the progression of vascular calcification [46,51].

### 2.3. Oxidative Stress

OS in CKD significantly contributes to kidney function decline and the progression of atherosclerosis. OS is a consequence of impaired renal antioxidant function, diminished elimination of pro-oxidant factors and increased proinflammatory processes with enhanced mobilization of immune cells [4]. Elevated levels of OS indicators such as mitochondrial superoxide and oxidized low-density lipoprotein cholesterol (LDL), along with increased serum homocysteine concentrations, as well as a superoxide dismutase (SOD) and glutathione (GSH) deficiency, are observed in CKD and increase with the decline in renal function [52]. A comparative study involving 167 patients with CKD revealed a significant elevation in lipid oxidative product levels among patients with stage 4 CKD, as well as those receiving HD and PD, with increases of 1.5-fold, 2.4-fold, and 2-fold compared to CKD stage 1 patients, respectively. The levels of SOD and GSH were analyzed similarly and were found to be significantly lower in the dialysis and CKD4 groups compared to patients with stage 1 CKD [53]. The interaction between dialysis membranes and blood may lead to the release of ROS and oxidizing agents, which could contribute to the oxidation of lipids, proteins, and nucleic acids [54]. This hypothesis was further supported by a study evaluating plasma malondialdehyde (MDA) levels, as a marker of lipid peroxidation, together with SOD and glutathione peroxidase (GPx) activities in HD patients before and after dialysis. The authors demonstrated that post-dialysis samples were characterized by significantly reduced SOD and GPx activities and elevated MDA levels, indicating enhanced OS following HD [55,56]. However, it is important to note that these findings were not consistently confirmed across all studies investigating these alterations [57]. Evidence from a mouse model indicates that increased macrophage myeloperoxidase (MPO) activity in the setting of CKD accelerates atherosclerosis progression and promotes MPO-derived oxidant-mediated damage of vascular wall proteins [58,59]. According to numerous studies, elevated serum uric acid levels constitute a prognostic factor for the presence and progression of atherosclerotic disease across the vascular bed [60,61,62,63]. Other factors promoting atherosclerotic plaque formation in CKD include oxidized thiols, vitamin C deficiency, and uremic toxin-induced dysfunctional form of eNOS [52]. Regardless of uric acid levels, elevated xanthine oxidase (XO) activity was found to be an independent predictor of cardiovascular risk in patients with CKD [64]. Endothelial NO is a vasoprotective mediator that maintains vascular tone by inducing vasodilation. In pathological states such as CKD, eNOS may become disrupted and, instead of generating NO, trigger the production of ROS. As a consequence, ROS further promote and sustain impaired eNOS function, thereby enhancing OS and exacerbating vascular dysfunction [52].

Anemia frequently occurs in CKD and intravenous iron supplementation is one of the therapeutic strategies [65]. Yet in CKD, iron metabolism has evolved from a simple focus on deficiency to a complex dysregulation driven by inflammation. Chronic low-grade inflammatory state in CKD upregulates hepcidin, favors iron sequestration in macrophages and induces functional iron deficiency [66,67]. At the tissue level, excess labile iron under OS can trigger ferroptosis, thereby promoting kidney injury and vascular damage, whereas macrophage iron deficiency may exacerbate OS, inflammation and kidney tissue fibrosis [68,69,70]. Therefore, iron imbalance, both deficiency or overload, is associated with the progression of atherosclerosis and vascular calcification, partly by promoting ROS synthesis, but also by impairing endothelial function, and modulating VSMC phenotype [71,72].

Clinically, intravenous iron and agents targeting the hypoxia-inducible factor-prolyl hydroxylase domain pathway improve anemia management and may support cardiac function, whereas iron overload and the formation of non-transferrin-bound iron raise concerns regarding endothelial damage and thrombotic risk. Studies indicate that iron supplementation leads to endothelial dysfunction and ROS production in patients with CKD, thereby accelerating atherogenesis [65,73,74,75]. Thus, in CKD and cardiorenal disease, iron represents a double-edged therapeutic modality, whose overall effect—beneficial or detrimental—is dictated by the balance of iron availability, inflammatory status, and OS, highlighting the necessity of individualized management strategies [76].

### 2.4. Endothelial Dysfunction

Endothelial dysfunction is one of the main factors in CKD that contribute to accelerated atherosclerosis in this patient population. Endothelial dysfunction occurs even at early stages of CKD, and its severity increases with the progression of renal impairment.

The vascular wall is classically organized into three layers arranged from the outermost to the innermost: the tunica adventitia, tunica media, and tunica intima. The media is composed predominantly of VSMC, whereas the intima is lined by endothelial cells resting on a subendothelial matrix. Physiologically, vascular homeostasis depends on the coordinated interaction between endothelial cells and VSMC, which together regulate vascular tone, permeability, inflammation, thrombosis, and structural remodeling. Disruption of this balance promotes vascular pathology, including atherosclerotic plaque formation, medial remodeling, and vascular calcification [77,78,79].

Vasomotor function refers to the ability of blood vessels to constrict or relax in response to mechanical and biochemical stimuli. Although the final effector response is mediated by vascular smooth muscle, vasomotor regulation may be either endothelium-dependent or endothelium-independent [80]. These responses are tightly linked to intracellular calcium signaling in smooth muscle cells: an increase in cytosolic calcium promotes vasoconstriction, whereas a reduction in calcium availability favors vasorelaxation [80]. Endothelium-dependent vasoconstriction reflects increased endothelial generation of contractile mediators including endothelin-1, ROS, angiotensin II, and arachidonic acid derivatives, whereas endothelium-dependent vasodilation is mediated primarily by NO, prostacyclin (PGI_2_), and endothelium-derived hyperpolarizing factor [80,81,82,83,84,85]. In CKD, endothelial homeostasis progressively shifts toward a vasoconstrictive, pro-oxidative, pro-inflammatory, and prothrombotic phenotype [86]. A central abnormality is the reduced bioavailability of NO, driven by OS, chronic inflammation, accumulation of endogenous nitric oxide synthase inhibitors such as asymmetric dimethylarginine (ADMA), and retention of uremic toxins. In parallel, the relative excess of vasoconstrictor and pro-inflammatory mediators further amplifies endothelial dysfunction [78,79,87,88,89,90]. Clinical studies support a graded association between renal dysfunction and endothelial impairment. In the study by Yilmaz et al. involving 159 individuals with CKD, worsening CKD stage was associated with progressively lower flow-mediated dilation, while ADMA and OS markers were significantly increased compared with healthy controls. Importantly, both were independent determinants of impaired endothelial function [88]. This relationship between declining estimated glomerular filtration rate (eGFR) and worsening endothelial function was confirmed by subsequent reports [78,88,89,91].

A persistent pro-inflammatory state further aggravates endothelial injury in CKD. IL-6 decreases endothelial eNOS expression, reduces adiponectin signaling, and increases ROS, thereby favoring vasoconstriction and atherogenesis. In addition, IL-1 and TNF-α activate endothelial cells and upregulate adhesion molecules such as intercellular adhesion molecule 1 (ICAM-1) and vascular cell adhesion molecule 1 (VCAM-1), enhancing monocyte adhesion and transendothelial migration into the intima. Within the vascular wall, monocyte-derived macrophages internalize modified lipoproteins through scavenger receptors, promoting foam cell formation and early atherogenesis [10,84,92,93,94,95,96,97]. Experimental data further show that uremic serum directly damages endothelial cells, increases apoptosis, upregulates ICAM-1 expression, and promotes excessive neutrophil extracellular trap formation, thereby amplifying inflammation, OS, endothelial permeability, and vascular injury [98].

Additional CKD-related factors further compromise endothelial function. Vitamin D deficiency, highly prevalent in CKD, was associated with impaired endothelial function and lower flow-mediated dilatation (FMD). Experimental data suggest that active vitamin D may exert vasculoprotective effects by increasing NO bioavailability and attenuating oxidative and inflammatory signaling, including NF-κB-related pathways. Interventional evidence is suggestive but not fully uniform: short-term vitamin D receptor activation or vitamin D supplementation improved FMD in some trials, and meta-analytic data indicate an overall favorable effect on endothelial function, although heterogeneity between studies remains substantial [99,100,101,102,103].

Anemia may also aggravate endothelial dysfunction through chronic tissue hypoxia, OS, and impaired vascular adaptation. In patients with non-dialysis CKD and anemia, erythropoietin therapy was associated with a significant improvement in endothelial function, including an absolute increase in FMD of approximately 10% [104].

Proteinuria should be interpreted not only as a marker of glomerular barrier injury but also as a manifestation of systemic endothelial damage. Increasing evidence links albuminuria with endothelial glycocalyx disruption, microvascular permeability, inflammation, OS, and higher cardiovascular risk. Thus, persistent albuminuria in CKD is better viewed as both a marker and an amplifier of widespread vascular injury rather than a purely renal phenomenon [78,79]. According to European Society of Cardiology guidelines on cardiovascular disease and progression in clinical practice (2021) [105] for the purpose of estimating cardiovascular disease risk categories in CKD, in addition to eGFR values, only the albumin-to-creatinine ratio is used. Thus, the degree of albuminuria was recognized as the only biomarker of cardiovascular risk in patients with CKD [105].

Endothelial dysfunction in CKD is also associated with impaired antithrombotic and fibrinolytic capacity. Patients with CKD, particularly those receiving HD, exhibit an elevation in prothrombotic factors, such as plasminogen activator inhibitor-1, along with impaired endothelial release of tissue-type plasminogen activator. This shift may reduce local protection against arterial thrombosis and facilitate thrombus formation on disrupted atherosclerotic plaques [106,107,108].

Recently, microRNA-92a (miR-92a) has emerged as a potentially relevant mediator of endothelial dysfunction in CKD. Experimental and translational data indicate that miR-92a reduces eNOS-derived NO bioavailability, enhances endothelial innate immune activation, and promotes vascular inflammation. In CKD, circulating miR-92a levels are elevated and inversely associated with eGFR, suggesting a close relationship with renal function decline. Furthermore, OS can upregulate endothelial miR-92a via sterol regulatory element-binding protein 2 transactivation, providing a mechanistic link between the uremic milieu and endothelial injury [109,110].

### 2.5. Advanced Glycation End Products

AGEs represent an extensive spectrum of compounds formed as a result of non-enzymatic glycation and oxidation of proteins and lipids. While circulating low-molecular-weight AGEs are primarily eliminated by renal clearance in individuals with preserved renal function, the accumulation of AGEs in CKD results from both impaired renal elimination and increased formation triggered by OS and inflammatory stress. Furthermore, increasing evidence indicates that carbonyl stress, especially related to reactive dicarbonyls like methylglyoxal, significantly contributes to the excessive formation of AGEs in CKD [111,112,113].

The clinical relevance of tissue AGE burden extends beyond elevated circulating concentrations. Elevated accumulation of AGEs has harmful cardiovascular effects via multiple complementary mechanisms.

Initially, AGEs establish stable cross-links with extracellular matrix proteins, such as collagen and elastin, in the vascular wall, which diminishes arterial elasticity and contributes to the progressive stiffening of arteries. AGE-modified collagen exhibit reduced susceptibility to degradation by metalloproteinases, potentially promoting the persistence of structural alterations [113,114,115].

Secondly, AGEs dysregulate endothelial homeostasis through impairment of NO signaling. AGEs do not operate through a singular pathway. They suppress eNOS expression while simultaneously increasing OS. The interaction of AGEs with the receptor for advanced glycation end products (RAGE) on endothelial cells initiates NF-κB-dependent proinflammatory signaling pathways, which increase the expression of adhesion molecules and cytokines, thus maintaining a proinflammatory and proatherogenic vascular environment [116,117,118].

AGEs also have a role in vascular calcification. Experimental studies indicate that the accumulation of AGEs enhances the osteogenic transdifferentiation of VSMC, elevates calcium deposition in the extracellular matrix, stimulates alkaline phosphatase activity, and induces apoptosis, thus promoting matrix mineralization and calcification. Additionally, AGE/RAGE signaling is associated with Wnt/β-catenin-mediated calcium deposition and influences autophagy and apoptosis-related phenotypic alterations in VSMC [119,120,121,122,123].

Both serum AGE concentrations and tissue AGE accumulation appear to be clinically meaningful in CKD. Research indicates that skin autofluorescence, utilized as a non-invasive proxy for tissue AGE burden, correlates with vascular calcification, arterial stiffness and the progression of macrovascular disease. Nonetheless, this technique should be regarded as an indirect indicator rather than a direct assessment of vascular calcification [124,125,126].

Collectively, these findings suggest that in CKD, the accumulation of AGEs should be regarded not solely as a result of diminished renal clearance but as an element of the extensive uremic, oxidative, and carbonyl stress environment that fosters endothelial dysfunction, arterial stiffening, vascular calcification, and cardiovascular risk [112,113].

### 2.6. Uremic Toxins

Progressive decline in renal function leads to the accumulation of numerous metabolites as a result of reduced renal clearance and their increased synthesis. Uremic toxins, including indoxyl sulfate (IS), p-cresyl sulfate (PCS), and trimethylamine N-oxide (TMAO), may contribute to inflammation and vascular injury in CKD. Their potential effects involve activation of NF-κB signaling, leading to enhanced OS and increased expression of adhesion molecules in endothelial cells, which in turn promote leukocyte recruitment, aggravate endothelial dysfunction, and accelerate atherosclerotic processes in the coronary arteries (Table 1) [90,127]. It should be emphasized that most mechanistic evidence derives from in vitro studies and animal models, which frequently employ toxin concentrations exceeding the free circulating levels observed in patients. Therefore, while these studies provide valuable mechanistic insights, they do not constitute direct evidence of such effects in humans and should be interpreted with caution. Moreover, although clinical association studies have linked higher circulating levels of protein-bound uremic toxins with adverse cardiovascular outcomes in CKD patients, a definitive causal relationship at clinically relevant concentrations has yet to be established [128,129,130].

### 2.7. Modification of Lipoproteins

Patients with CKD exhibit characteristic abnormalities of lipid metabolism, although the pattern and severity of these disturbances vary according to CKD stage and renal replacement modality. Clinically, the most typical findings include elevated serum triglyceride (TG) concentrations and increased levels of TG-rich lipoproteins, such as very-low-density lipoproteins (VLDL), chylomicrons, and intermediate-density lipoproteins, whereas high-density lipoprotein (HDL) cholesterol is frequently reduced. In contrast, LDL concentrations are often unchanged or only modestly altered, meaning that conventional lipid measurements may underestimate the true atherogenic burden in CKD [4,154].

Clinical studies indicate that hypertriglyceridemia is a central component of CKD-associated dyslipidemia. In a comparative kinetic study, patients with non-dialysis CKD and patients treated with PD were matched with controls for age and body mass index. HDL concentrations were similar in the non-dialysis CKD and control groups, whereas in PD patients they were approximately 30% lower than in matched controls. In the non-dialysis CKD group, total plasma TG, VLDL-TG, and VLDL-apolipoprotein B-100 (apoB-100) concentrations were 10–30% higher than in controls, but these differences were not statistically significant. By contrast, all three parameters were more than two-fold higher in PD patients than in their matched controls. Importantly, the secretion rates of VLDL-TG and VLDL-apoB-100 were not increased, supporting the conclusion that hypertriglyceridemia in CKD results predominantly from impaired plasma clearance of TG-rich lipoproteins rather than from hepatic overproduction alone [155].

Clinical and experimental data further suggest that abnormalities of HDL in CKD are not limited to reduced circulating HDL levels but also involve profound disturbances in HDL maturation and function. In the uremic state, lower HDL concentrations were linked to reduced hepatic synthesis and increased catabolism of apolipoprotein A-I (ApoA-I), as well as to downregulation of lecithin–cholesterol acyltransferase, which impairs HDL maturation and reverse cholesterol transport [156,157]. Experimental in vitro data additionally suggest that the uremic factor or factors responsible for suppression of ApoA-I expression are not effectively removed by HD [156]. Experimental proteomic studies also showed that the molecular composition of HDL is markedly altered in CKD. HDL particles become depleted of several cardioprotective components, including ApoA-I, apolipoprotein M, and paraoxonase-1, while accumulating proteins and solutes associated with inflammation and dysfunction, such as serum amyloid A, surfactant protein B, apolipoprotein C-III, and symmetric dimethylarginine [158,159,160]. These compositional changes are associated with impaired cholesterol efflux capacity and with the loss of the anti-inflammatory, antioxidative, and endothelial-protective properties of HDL. In some experimental settings, uremic HDL acquires pro-inflammatory activity instead [159,160,161]. Post-translational modifications may further aggravate these abnormalities [158].

Clinical observations supported by mechanistic studies suggest that HDL dysfunction begins early in CKD and becomes more pronounced as renal function declines. In children with CKD, HDL dysfunction was detectable already in earlier disease stages and worsened with decreasing eGFR, indicating that qualitative HDL abnormalities may precede end-stage kidney disease [161]. However, these findings primarily concern HDL function rather than serum HDL concentration alone and should therefore not be interpreted as evidence that all classical lipid abnormalities are already fully established in early CKD [161].

In dialysis populations, clinical studies indicate that HDL abnormalities persist in both HD and PD, although the pattern differs somewhat between modalities. In both HD and PD, HDL particles show a more pro-inflammatory composition, reduced ApoA-I, apolipoprotein A-II, and paraoxonase-1 content, and enrichment with TG. Functional impairment appears to be more pronounced in HD, where HDL demonstrates greater reduction in cholesterol efflux capacity, anti-inflammatory activity, and antiapoptotic properties, together with a shift toward smaller HDL3 particles [162]. In PD, the most prominent abnormality appears to be markedly reduced HDL-associated paraoxonase activity, whereas impairment of anti-inflammatory HDL function seems less severe than in the HD population [162].

Experimental studies also indicate that structurally modified HDL may directly contribute to vascular injury. Oxidized and carbamylated HDL were shown to impair endothelial repair, promote OS and inflammatory signaling and adversely affect vascular homeostasis. In end-stage renal disease, carbamylated HDL specifically inhibited endothelial cell proliferation, migration, and re-endothelialization capacity, supporting the concept that dysfunctional HDL in CKD may contribute to atherogenesis rather than exerting its usual vasculoprotective effects [163].

The contribution of LDL to atherosclerosis in CKD is less related to absolute LDL concentration than to qualitative modification of LDL particles. Mechanistically, atherosclerosis develops through lipid accumulation and inflammatory cell infiltration within the subendothelial space. Modified LDL particles, particularly oxidized LDL (oxLDL), are taken up by macrophages through scavenger receptors, especially SR-A and CD36, which promotes foam-cell formation. OxLDL is increased in the setting of OS and has been reported to be elevated in uremia. After foam-cell death, released lipid material contributes to formation of the necrotic core of the atherosclerotic plaque [164]. Clinical studies suggest that expression of the scavenger receptor CD36 differs across CKD populations. In one study, monocyte CD36 expression was significantly higher in both HD and PD patients than in controls, whereas in predialysis patients the increase exceeded 15% but did not reach statistical significance. In the same study, statin therapy reduced monocyte CD36 expression in dialyzed patients, with the effect being particularly pronounced in PD. These findings are clinically interesting but should be interpreted cautiously, as they derive from a relatively small study group and concern an intermediate mechanistic marker rather than hard cardiovascular outcomes [165].

Experimental and translational studies demonstrated that elevated urea directly promotes the formation of carbamylated LDL (cLDL), which is increased in the circulation of uremic patients [166]. Carbamylation of LDL results from nonenzymatic chemical modification of apolipoprotein B by cyanate derived from urea, rather than by undefined ‘uremic toxins’ in general [166]. Experimental studies further show that cLDL exerts multiple proatherogenic effects, including increased monocyte adhesion to endothelial cells through upregulation of ICAM-1 and VCAM-1, endothelial cytotoxicity, and promotion of VSMC proliferation [167,168].

A single LDL particle may undergo both oxidation and carbamylation, forming carbamylated-oxidized LDL (coxLDL), which appears to be particularly proatherogenic in experimental models. Although these two modifications may partially compete, they can coexist within the same LDL particle [164]. Compared with cLDL or oxLDL alone, coxLDL was shown experimentally to induce greater endothelial toxicity, stronger foam-cell formation, and more pronounced macrophage injury, largely through scavenger receptor-mediated pathways, particularly CD36 [164].

The clinical significance of these lipid abnormalities in CKD should not be overstated. Although numerous mechanistic and observational studies support a proatherogenic role of dysfunctional HDL and modified LDL in CKD, not all clinical studies have demonstrated a clear association between individual oxidized lipid markers and cardiovascular outcomes [169]. Therefore, the available evidence suggests that cardiovascular risk in CKD is driven not only by quantitative changes in lipid concentrations, but also by substantial qualitative alterations in lipoprotein structure and function. However, the clinical utility of many of these biomarkers remains uncertain [169].

### 2.8. Gut–Kidney–Heart Axis

In the past few years, gut microbiota was extensively explored in multiple disease models, including CKD and CVD [170,171]. Patients with impaired kidney function commonly exhibit gastrointestinal dysfunction, gut dysbiosis, and reduced microbial diversity. These alterations favor a shift toward proteolytic fermentation and the intestinal generation of gut-derived uremic solutes, including indole- and phenol-derived compounds such as IS and PCS, as well as trimethylamine, which is subsequently oxidized in the liver to TMAO [172,173]. In parallel with the expansion of proteolytic taxa, CKD is associated with depletion of saccharolytic, short-chain fatty acid (SCFA)-producing bacteria, including butyrate-producing genera such as *Roseburia* and related taxa, together with a measurable reduction in fecal SCFAs, particularly butyrate [172,174]. This shift is biologically important because it reflects a transition from carbohydrate fermentation toward proteolytic fermentation, favoring the generation of indole- and phenol-derived solutes [175,176,177].

As renal clearance diminishes with the course of CKD, several microbiota-derived compounds accumulate in the bloodstream [178]. Retention of urea in CKD increases its luminal intestinal concentration, and bacterial urease converts urea into ammonia and ammonium hydroxide, which disrupt epithelial tight junction proteins including occludin, claudin-1, and zonula occludens 1 (ZO-1), thereby increasing intestinal permeability [179,180,181]. Additionally, CKD patients are frequently exposed to factors that may further promote dysbiosis and ‘leaky gut’, including reduced dietary fiber intake, prolonged colonic transit and polypharmacy. The contribution of medication-related microbiome alterations is supported by broader human data, whereas the direct mechanistic role of metabolic acidosis remains biologically plausible but less firmly established [182,183].

Increased low-grade endotoxemia was detected across the spectrum of CKD, although no clear linear relationship between eGFR and circulating endotoxin levels was demonstrated. Importantly, serum endotoxin concentrations were markedly higher in patients receiving dialysis than in non-dialyzed CKD patients [184]. In line with this, subsequent observational data suggested that the mode of renal replacement therapy may itself influence endotoxemia, with HD patients exhibiting higher endotoxin levels than patients treated with PD or those after kidney transplantation [185].

Together, dysbiosis and increased intestinal permeability may facilitate the translocation of lipopolysaccharide (LPS) and other gut-derived microbial products into the circulation. These products activate innate immune signaling through CD14 and Toll-like receptor 4 (TLR4), thereby amplifying systemic inflammation [186,187,188]. Clinical studies support this concept. Circulating endotoxin and soluble CD14 (sCD14) levels are increased in CKD, particularly in patients receiving dialysis, and higher sCD14 concentrations are associated with inflammation, mortality, and cardiovascular risk. However, some of these associations are attenuated after adjustment for renal disease severity or proteinuria, indicating that residual confounding may still be present [184,186,189]. Circulating endotoxin-related markers also vary according to CKD severity and dialysis status. In patients with non-dialysis CKD, plasma sCD14 increases as kidney function declines and is associated with higher mortality rate and cardiovascular disease (CVD), although its association with CKD progression is attenuated after adjustment for proteinuria. In long-term HD patients, elevated sCD14 correlates with inflammatory activation, including higher TNF-α and IL-6 levels, and independently predicts mortality. More recent data from HD cohorts further support associations between higher sCD14, inflammation, subclinical atherosclerosis, and mortality [190]. Activation of the CD14/TLR4 axis promotes downstream NF-κB signaling and inflammasome-related cytokine responses, including increased IL-1β and IL-6 production, thereby sustaining low-grade systemic inflammation. In vascular cells, LPS/TLR4 signaling also enhances OS and increases the expression of adhesion molecules, supporting leukocyte recruitment and a pro-inflammatory vascular phenotype [191,192]. Experimental data further support a pathogenic role of this pathway. CKD animals exhibit disruption of intestinal tight junction proteins (occludin, claudins, ZO-1), increased bacterial translocation and endotoxemia, whereas genetic disruption of TLR4 signaling attenuates NF-κB activation, cytokine release, renal fibrosis and CKD progression [179,180,181,193].

Recent work also strengthened the relationship between dysbiosis, endotoxin signaling, and CKD-associated vascular injury. In a 2024 mechanistic study, increased abundance of *Prevotella copri* promoted vascular calcification in CKD through LPS-driven activation of TLR4, NF-κB, and NLRP3 signaling, providing more direct evidence that gut microbial products may contribute to cardiovascular pathology in CKD [194].

CKD-associated dysbiosis also affects bile acid metabolism. Elevated circulating deoxycholic acid was associated with coronary artery calcification in patients with moderate CKD, and experimental data suggest that deoxycholic acid may directly exacerbate vascular calcification through stress-mediated signaling [195,196,197].

Finally, TMAO remains an important candidate mediator linking the gut microbiota with cardiovascular risk in CKD. Although whether TMAO is a causal toxin or, at least in part, a marker of impaired renal clearance remains debated, recent meta-analytic data indicate that higher circulating TMAO is associated with increased all-cause and cardiovascular mortality in patients with CKD [172,198].

**Figure 2:**
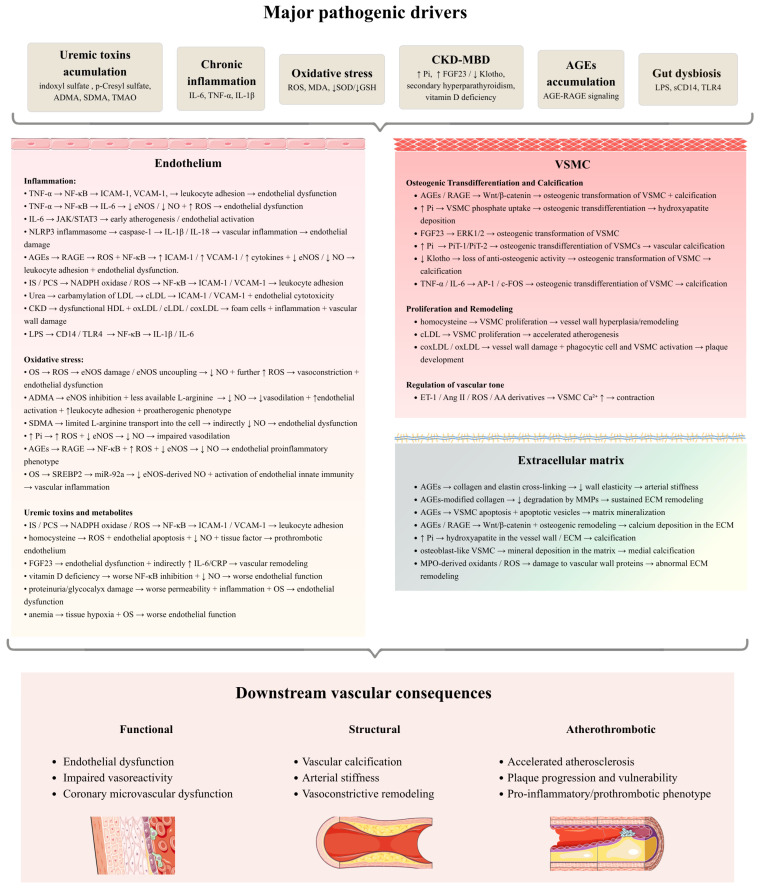
Major pathogenic drivers of CKD-associated vascular injury and coronary atherosclerosis. ADMA, asymmetric dimethylarginine; AGEs, advanced glycation end products; Ang II, angiotensin II; AA, arachidonic acid; AP-1, activator protein 1; c-FOS, cellular Fos proto-oncogene protein; Ca^2+^, calcium ion; cLDL, carbamylated low-density lipoprotein; CD14, cluster of differentiation 14; CKD, chronic kidney disease; CKD-MBD, chronic kidney disease–mineral and bone disorder; coxLDL, carbamylated oxidized low-density lipoprotein; CRP, C-reactive protein; ECM, extracellular matrix; eNOS, endothelial nitric oxide synthase; ERK1/2, extracellular signal-regulated kinase 1/2; ET-1, endothelin 1; FGF23, fibroblast growth factor 23; GSH, glutathione; HDL, high-density lipoprotein; ICAM-1, intercellular adhesion molecule 1; IL-1β, interleukin 1 beta; IL-6, interleukin 6; IL-18, interleukin 18; IS, indoxyl sulfate; JAK, Janus kinase; LPS, lipopolysaccharide; MDA, malondialdehyde; miR-92a, microRNA-92a; MMPs, matrix metalloproteinases; MPO, myeloperoxidase; NADPH, reduced nicotinamide adenine dinucleotide phosphate; NF-κB, nuclear factor kappa B; NLRP3, NLR family pyrin domain containing 3; NO, nitric oxide; OS, oxidative stress; oxLDL, oxidized low-density lipoprotein; PCS, p-cresyl sulfate; Pi, inorganic phosphate; PiT-1, phosphate transporter 1; PiT-2, phosphate transporter 2; RAGE, receptor for advanced glycation end products; ROS, reactive oxygen species; SDMA, symmetric dimethylarginine; SOD, superoxide dismutase; SREBP2, sterol regulatory element-binding protein 2; STAT3, signal transducer and activator of transcription 3; TLR4, toll-like receptor 4; TMAO, trimethylamine N-oxide; TNF-α, tumor necrosis factor alpha; VCAM-1, vascular cell adhesion molecule 1; VSMC, vascular smooth muscle cell; Wnt, Wingless-related integration site signaling pathway; ↑, increase or upregulation; ↓, decrease or downregulation. Images adapted from Servier Medical Art (https://smart.servier.com/), licensed under CC BY 4.0 (https://creativecommons.org/licenses/by/4.0/, accessed on 13 April 2026).

CKD-specific pathogenic components interact at several levels, resulting in self-reinforcing development and progression of atherosclerosis. Figure 2 presents a summary of the most important mechanisms.

## 3. Atherosclerosis with and Without CKD

### 3.1. Composition of Atherosclerotic Plaques

Numerous studies indicate that the morphology of coronary atherosclerotic plaques is different in CKD patients compared to those with normal kidney function, although these differences should be interpreted with caution. Optical coherence tomography (OCT), virtual histology intravascular ultrasound (VH-IVUS), integrated backscatter intravascular ultrasound (IB-IVUS), computed tomography (CT) and histopathological autopsy studies do not assess identical structural features and differ substantially in spatial resolution, tissue discrimination, and definitions of plaque vulnerability [199,200,201,202,203].

OCT analyses showed that patients with CKD tend to exhibit a higher plaque lipid burden, reflected by a wider lipid arc, longer lipid-rich plaques and a higher lipid index than patients without renal dysfunction. The same OCT study demonstrated that cholesterol crystals were more frequently detected in plaques from patients with CKD than in those from individuals without CKD, supporting the concept of a more advanced plaque phenotype in the CKD population [199]. The higher frequency of cholesterol crystals in CKD may indicate greater plaque complexity, as they are generally linked to advanced fibroatheroma, extensive necrotic cores, and intraplaque bleeding, rather than being a mere independent imaging observation [204].

Evidence from compositional intravascular ultrasound studies is broadly consistent with this interpretation. IB-IVUS and VH-IVUS studies showed a significantly higher proportion of lipid volume and a lower proportion of fibrous tissue in coronary plaques of patients with CKD compared to those without decreased kidney function [203,205,206]. Hayano et al. demonstrated that lower eGFR was associated with higher lipid content and lower fibrous content in coronary plaques, suggesting increased plaque vulnerability with worsening renal function [206]. Similarly, Ogita et al. reported that worsening renal function in diabetic patients was associated with an increase in necrotic core volume within non-culprit coronary lesions [205].

Histopathological studies further support the presence of a more advanced and biologically active plaque phenotype in CKD [201,207,208]. Autopsy data from the Japanese study conducted in Hisayama showed that declining kidney function was associated not only with more severe coronary atherosclerosis and calcification, but also with greater neovascularization and a higher prevalence of intraplaque hemorrhage [208].

In CKD, the inflammatory milieu promotes expansion of pro-atherogenic CD16-positive monocyte subsets, enhances endothelial activation, and facilitates monocyte-endothelial interactions through the CX3CR1/CX3CL1 axis, thereby favoring leukocyte recruitment to the vascular wall [209]. Experimental studies further indicate that renal dysfunction accelerates atherogenesis and is associated with increased macrophage-rich atherosclerotic lesion burden [210]. After entering the vascular intima, macrophages internalize modified lipoproteins, including oxLDL, and undergo transformation into foam cells, which contribute to plaque progression [211]. Significantly, as demonstrated in mouse models, CKD not only facilitates the progression of atherosclerosis but also impairs the regression of pre-existing atherosclerotic plaques, indicating a compromised resolution of vascular inflammation [212]. A molecular interpretation is that impaired renal function disrupts macrophage lipid homeostasis by inhibiting ATP-binding cassette transporter A1-mediated cholesterol export, thereby increasing intracellular lipid buildup and foam cell formation [211]. In addition, coronary plaques from patients with CKD were found to contain more inflammatory cells, including macrophages, mast cells, and dendritic cells, indicating intensified local inflammatory activity within the atherosclerotic lesion [207,213].

Nevertheless, the available evidence does not support the unequivocal conclusion that CKD uniformly produces thinner fibrous caps or a higher prevalence of thin-cap fibroatheroma in all clinical settings [199,200]. The PROSPECT analysis did not demonstrate a significant difference in VH-IVUS-defined thin-cap fibroatheroma prevalence between patients with and without CKD [200].

Likewise, OCT analyses did not reveal a significant difference in mean fibrous cap thickness between CKD and non-CKD groups [199]. Moreover, coronary CT angiography in patients with mild CKD suggested that although stenotic disease burden was greater, the prevalence of high-risk plaque was not significantly different from that in patients without CKD [202]. These inconsistencies may partly result from methodological differences between imaging modalities, but they also likely reflect the marked heterogeneity of the studied CKD populations. Specifically, some studies included patients with mild CKD, others examined acute coronary syndrome (ACS) cohorts, while others focused on patients with diabetes mellitus and hypertension. Additionally, some reports were based on elderly autopsy material or dialysis populations [199,200,202,203,208].

Therefore, the most balanced interpretation is that CKD is generally associated with a more lipid-rich, more inflamed, and in some cohorts more hemorrhagic or neovascularized coronary plaque phenotype, whereas evidence concerning uniformly thinner fibrous caps and a consistently higher prevalence of thin-cap fibroatheroma remains inconclusive [199,200,203,208].

### 3.2. Calcification

Patients with CKD have coronary atherosclerotic lesions marked by an elevated calcific burden, with coronary artery calcification potentially being evident and progressing in the initial stages of renal function deterioration [214,215,216]. Autopsy studies showed that, compared with non-uremic controls, patients with end-stage kidney disease more often exhibit heavily calcified coronary plaques and greater coronary medial thickness, suggesting that the morphology of coronary disease in CKD differs not only quantitatively but also qualitatively [214,217]. Intravascular ultrasound studies further indicate that declining eGFR is associated with a progressive increase in dense calcium within coronary culprit lesions, together with more advanced plaque morphology [218,219]. From a histopathological perspective, vascular calcification in CKD may engage either the intima, where it accompanies atherosclerotic plaque evolution, or the media, where it reflects arteriosclerotic remodeling [220,221]. Although coronary arteries are muscular vessels and therefore generally exhibit less medial calcification than large elastic arteries, medial coronary calcification is observed more often in CKD than in individuals without renal disease [217,221]. Accordingly, arterial medial calcification is especially prevalent in patients receiving HD [222]. This distinction is clinically important as medial arterial calcification does not primarily cause luminal stenosis but instead promotes arterial stiffening with further hemodynamic consequences [220,223].

In CKD, mineral deposition along the elastic lamellae increases pulse-wave velocity, reduces arterial compliance, and contributes to systolic hypertension and widened pulse pressure [222,224,225]. Increased central arterial stiffness also lowers diastolic aortic pressure and advances wave reflection, thereby reducing the diastolic perfusion gradient that is required for coronary blood flow and functionally impairing coronary flow reserve even in the absence of critical epicardial stenosis [226,227]. Mechanistically, this process is not passive mineral encrustation but an actively regulated vasculopathy in which hyperphosphatemia, uremic toxins, Klotho deficiency, and osteogenic transdifferentiation of VSMC promote calcium-phosphate deposition [31,46,50,228].

Calcifications in atherosclerotic plaques in CKD may present as small microcalcifications (0.5–15.0 μm) within the lipid core, larger foci referred to as spotty calcifications, or extensive sheet-like deposits (>3 mm) [229,230]. Fracture of the calcified sheath results in the formation of nodular calcifications, which represent a distinctive high-risk lesion phenotype in which eruptive nodular calcium disrupts the fibrous cap and protrudes into the lumen, favoring fibrin deposition, endothelial loss, and thrombus formation [230]. Although calcified nodules account for only a minority of acute coronary thrombotic events overall, they appear to be particularly relevant in older patients and in those with CKD or dialysis dependence [231,232,233].

Patients treated with dialysis exhibit a unique calcific phenotype of restenotic tissue following coronary stenting, in addition to native plaque calcification. Histopathological analysis of directional coronary atherectomy specimens from in-stent restenosis lesions revealed that in-stent calcified nodules were significantly more prevalent in patients undergoing dialysis (75% vs. 5% in non-dialysis patients). Conversely, lipid-rich in-stent plaque was not present in dialysis patients (0% vs. 43%). Calcified nodules in the dialysis group were predominantly identified within the first year following stent implantation, indicating a rapid and mechanistically unique pathway of restenosis in end-stage kidney disease. These findings complement intravascular and autopsy observations of extensive coronary calcification in CKD and support the concept that coronary disease in dialysis patients is strongly influenced by mineral-disorder-associated vascular osteogenic remodeling and calcific injury [36]. Recent clinical data further support the adverse prognostic significance of calcified nodules in end-stage renal disease on dialysis [231].

In addition to the coronary circulation changes, calcification of major peripheral and central arteries, especially the aorta, is prevalent in CKD and significantly contributes to systemic arterial stiffness and ventricular–vascular uncoupling [140,222].

Importantly, newer prospective cohort data show that progression of coronary artery calcification in CKD is not merely an imaging marker but is associated with a higher risk of atherosclerotic cardiovascular events and all-cause mortality [234].

### 3.3. Stability and Susceptibility to Fracture

Large, confluent calcifications within atherosclerotic plaques are generally associated with a more advanced and often more stable plaque phenotype, whereas small, superficial, or spotty calcifications are more closely linked to plaque vulnerability and rupture-prone morphology [235,236]. Accordingly, extensive calcification is more commonly observed in older patients with advanced and diffuse CAD, while smaller calcium deposits tend to cluster in lesions with greater inflammatory activity and a more rupture-prone phenotype [237,238].

In CKD, however, coronary atherosclerosis is shaped not only by accelerated calcification but also by chronic inflammation, OS, endothelial dysfunction, and profound disturbances of mineral metabolism, all of which may modify plaque biology [218,239,240]. For this reason, extensive calcification in CKD should not be interpreted as unequivocal evidence of plaque stability, because heavily calcified lesions may coexist with lipid accumulation, macrophage infiltration, neovascularization, and morphologic complexity [213,218,241]. In an OCT study by Kato et al., plaque disruption was observed more frequently in patients with CKD than in those without CKD, supporting the concept that renal dysfunction is associated with more complex coronary plaque morphology [199]. Other primary imaging studies are directionally consistent with this interpretation, showing that worsening renal function is associated with greater plaque burden, longer lesions, larger rupture cavities, and more frequent thrombus in acute myocardial infarction [242].

In the general population, ACS most commonly arises from plaque rupture, although plaque erosion may also serve as the underlying cause. It is characterized by thrombus formation over an eroded endothelial surface in the presence of an intact fibrous cap. While both mechanisms are recognized causes of ACS, it remains unclear whether CKD alters their relative prevalence [243,244,245]. The hypothesis that uremia may favor plaque erosion remains biologically plausible, but direct CKD-specific evidence is still insufficient to conclude that plaque erosion replaces plaque rupture as the dominant substrate of ACS in this population [244,246]. Indeed, several original studies indicate that plaque rupture remains highly relevant in CKD, particularly in advanced renal dysfunction and end-stage kidney disease [218,241,242]. For example, Sugiyama et al. reported that patients with end-stage kidney disease had thinner fibrous caps, a larger calcification arc, and a higher prevalence of plaque rupture than patients without end-stage kidney disease [241]. Similarly, histopathologic work showed that coronary plaques in CKD may contain larger necrotic cores and more frequent plaque rupture, further arguing against an overly simplistic shift from rupture to erosion [218]. Therefore, the most accurate interpretation is that CKD is associated with a heterogeneous but distinctly high-risk coronary plaque phenotype characterized by advanced calcific remodeling together with persistent features of vulnerability [213,218,239,241].

Beyond plaque morphology, patients with CKD, especially those receiving HD, are also linked to enhanced coronary thrombogenicity, which may further amplify the risk of acute ischemic events and adverse outcomes after percutaneous coronary intervention [247,248]. In the OCT study by Konishi et al., intra-stent thrombus at mid-term follow-up after drug-eluting stent implantation was detected significantly more often in hemodialysis patients than in non-hemodialysis controls, and this association persisted after multivariable adjustment and propensity-score matching, suggesting that the excess thrombotic risk in hemodialysis is not explained solely by local vessel healing but also by systemic prothrombotic factors, including increased residual platelet reactivity [247].

Overall, the available primary evidence indicates that CAD in CKD combines extensive calcific remodeling, ongoing plaque vulnerability, and enhanced thrombogenicity, which together likely contribute to the exceptionally high cardiovascular risk observed in this population [199,218,241,242,247].

## 4. Microvascular Dysfunction

In CKD patients, myocardial ischemia cannot be explained solely by obstructive lesions in the epicardial coronary arteries [249]. Increasing evidence indicates that coronary microvascular dysfunction (CMD) contributes substantially to ischemic symptoms and adverse cardiovascular outcomes in this population, including patients without significant obstructive CAD [250,251]. Early clinical studies demonstrated that even mild renal insufficiency is associated with impaired coronary vasodilator capacity in patients with angiographically normal or with mild coronary artery stenosis, supporting the concept that abnormalities of the coronary microcirculation emerge early in the course of kidney dysfunction [249]. This concept was further reinforced by subsequent imaging studies showing that coronary microvascular impairment is already detectable in early CKD and is not confined to advanced stages of the disease [252,253].

The primary physiological indicator of CMD in CKD is reduced coronary flow reserve (CFR), reflecting impaired ability of the coronary circulation to augment blood flow during stress [252,254]. In a systematic review and meta-analysis by Jain et al., CFR was significantly reduced in patients with CKD compared to individuals without kidney function decrease, and this relationship was observed even in patients without obstructive CAD. Furthermore, a significant positive linear correlation was found between eGFR and CFR, indicating progressive impairment of coronary microvascular function with worsening renal function [252]. Beyond its diagnostic value, CFR appears to have major prognostic significance in CKD [250,255]. Positron emission tomography-based studies showed that lower CFR is associated with increased cardiovascular mortality across all CKD stages and remains prognostically informative even after adjustment for conventional cardiovascular risk factors [250]. More recent outcome data support the independent prognostic negative impact of depressed CFR in patients with CKD [255].

Importantly, coronary microvascular dysfunction in CKD should not be interpreted merely as an isolated abnormality of physiological indices such as CFR. Rather, it likely represents the functional expression of a broader systemic microvascular disease characterized by endothelial injury, impaired vasodilatory signaling, defective angiogenesis, and structural remodeling of the myocardial microcirculation [251,254]. This interpretation is supported by mechanistic and pathological studies showing that CKD is associated with widespread endothelial dysfunction and microvascular remodeling across multiple vascular beds [256]. One of the earliest documented microvascular abnormalities in CKD is injury of the endothelial glycocalyx, a structure essential for mechanotransduction, barrier integrity, and vascular homeostasis. Glycocalyx damage was demonstrated both in dialysis patients and in non-dialysis CKD populations [257,258]. Furthermore, the severity of glycocalyx injury correlates positively with uremic toxins and markers of endothelial dysfunction, supporting a direct link between the uremic milieu and impaired endothelial health. The strongest elevations in circulating markers of glycocalyx injury and endothelial cell damage were reported in patients receiving dialysis [259]. These observations support the hypothesis that coronary microvascular dysfunction in CKD develops on a background of generalized endothelial injury [257,258,259].

Additional mechanistic support comes from studies showing that CKD is associated with increased circulating inhibitors of angiogenesis and NO pathways, including factors that impair the proliferation and survival of cardiac endothelial cells and promote endothelial-to-mesenchymal transition. In a post-mortem and translational study, cardiac fibrosis increased by 12% in stage 3–4 CKD and by 77% in end-stage kidney disease, while microvascular density declined in parallel. These findings suggest that endothelial injury in CKD is not only functional, but also contributes to capillary rarefaction and progressive fibrotic remodeling of the myocardium [260]. Experimental models confirmed this structural paradigm. CKD was demonstrated to cause systemic microangiopathy, tissue hypoxia, and impaired angiogenesis in numerous microvascular systems [256]. In the heart tissue, animal studies demonstrated arteriolar wall thickening, capillary rarefaction, capillary-myocyte mismatch, and interstitial fibrosis, all of which would be expected to impair myocardial perfusion reserve and oxygen delivery [261]. Metabolic abnormalities of CKD, including hyperphosphatemia, may further aggravate post-coronary microvascular remodeling and myocardial fibrosis in experimental uremia [262]. Therefore, impaired coronary microvascular function may represent a mechanistic link between renal dysfunction, myocardial remodeling similar to heart failure with preserved ejection fraction (HFpEF), heart failure risk, and mortality. Both HFpEF and CKD share closely overlapping pathophysiological mechanisms, including arterial stiffness, coronary microvascular dysfunction, and persistent systemic inflammation. In this context, structural and functional alterations of the coronary microcirculation in CKD may play a central role in the development of non-obstructive myocardial ischemia. Impaired coronary microvascular function reduces myocardial perfusion reserve despite the absence of epicardial coronary artery stenosis, thereby promoting chronic subclinical ischemia. This mechanism may contribute to HFpEF-like myocardial remodeling observed in CKD, characterized by left ventricular hypertrophy, increased myocardial stiffness, and diastolic dysfunction, thereby linking renal dysfunction with adverse cardiac remodeling and outcomes. The overlap with HFpEF is further strengthened by evidence showing that CKD is characterized by persistent endothelial dysfunction, progressive vascular stiffening, and ongoing left ventricular mass progression with diastolic dysfunction, even in the moderate stages of kidney disease. These changes establish a pro-remodeling and pro-fibrotic cardiovascular milieu that predisposes to functional and structural cardiac impairment [251,263,264].

Notably, in a carefully selected cohort of patients with end-stage renal disease, coronary microvascular dysfunction remained common and was independently associated with lower hemoglobin levels, suggesting that anemia itself may be an underappreciated contributor to impaired coronary microvascular function in advanced kidney disease [265].

## 5. Conclusions

CKD significantly accelerates the progression of CAD. Alongside the common risk factors, CKD presents disease-specific mechanisms such as persistent low-grade inflammation, OS, mineral and bone disorders, endothelial dysfunction, uremic toxins, AGEs, and lipoprotein modification. These factors significantly contribute to a pro-atherogenic and prothrombotic environment. As a result, coronary atherosclerosis in CKD presents distinct characteristics compared to the ‘classic’ phenotype found in the general population. Atherosclerotic plaques in CKD frequently exhibit larger lipid-rich necrotic cores, heightened inflammatory cell infiltration, and a significant calcific burden, along with increased vulnerability characteristics such as cholesterol crystals and microdisruptions. In CKD, calcification primarily manifests as intimal calcification in the coronary arteries, which may occur alongside medial calcification that enhances arterial stiffness. Additionally, CKD characterizes a specific, accelerated type of CAD intensified by calcification and inflammation. This highlights the necessity for early diagnosis of cardiovascular risk in CKD patients to implement proper treatment procedures and preventive strategies that address both traditional and CKD-specific factors.

## Figures and Tables

**Table 1:** The impact of major uremic toxins on endothelial cells and their role in the development of atherosclerosis.

Uremic Toxin	Chemical Origin	Main Effects on Endothelial Cells	Contribution to Atherosclerosis
Indoxyl sulfate (IS)	Protein-bound uremic toxin; tryptophan-derived	↑ ROS production via NADPH oxidase ↑ activation of NF-κB and pro-inflammatory cytokines ↑ expression of adhesion molecules (VCAM-1, ICAM-1, E-selectin) ↓ eNOS expression and NO bioavailability ↑ endothelial senescence ↓ endothelial repair [131,132]	Promotes leukocyte adhesion and transmigration, vascular inflammation, endothelial barrier disruption and arterial stiffness. Contributes to calcification and accelerated plaque progression in CKD [90,131].
p-Cresyl sulfate (PCS)	Protein-bound, phenolic uremic toxin	↑ OS and pro-inflammatory signaling ↑ expression of adhesion molecules ↓ endothelial barrier integrity ↑ monocyte adhesion and migration (often synergistically with IS) [133,134]	Associated with endothelial dysfunction, vascular remodeling and media calcification. In dialysis patients, PCS is associated with vascular dysfunction and worse cardiovascular outcomes [127,131,134].
Asymmetric dimethylarginine (ADMA)	Endogenous methylated arginine; competitive inhibitor of NOS	↓ eNOS with further ↓ NO bioavailability ↑ OS ↑ pro-inflammatory and pro-thrombotic endothelial phenotype [135,136,137]	Associated with arterial stiffness and major cardiovascular events. Considered an independent cardiovascular risk factor in uremic patients [138,139,140].
Symmetric dimethylarginine (SDMA)	Endogenous dimethylated arginine; marker of reduced renal clearance; interferes with L-arginine transport	↓ NO synthesis by limiting L-arginine availability ↓ flow-mediated dilation ↓ microvascular reactivity in CKD ↑ endothelial dysfunction via oxidative and inflammatory pathways [136,141]	Correlates with reduced eGFR, impaired endothelial function and increased arterial stiffness in G3-G4 CKD stages, suggesting a role in early vascular damage and atherogenesis [79,142].
Homocysteine	Sulfur-containing amino acid	↑ OS and endothelial apoptosis ↓ NO availability ↑ expression of adhesion molecules and tissue factor ↓ anticoagulant properties of endothelium [143,144]	Favors LDL oxidation, smooth muscle cell proliferation and a pro-thrombotic state. Contributes to intimal hyperplasia and accelerated atherosclerosis in CKD [145,146,147].
Inorganic phosphate (Pi)	Small, water-soluble retained solute; increases in CKD due to reduced renal excretion (hyperphosphatemia)	↑ endothelial dysfunction, OS and apoptosis ↑ endothelial expression of calcification regulators and adhesion molecules ↑ endothelial microparticle release [148,149,150]	Central driver of vascular and valvular calcification, arterial stiffness and medial thickening. Plays a synergistic role with protein-bound toxins (IS, PCS) to promote calcific atherosclerosis in CKD [151].
Tryptophan metabolites (TRP)	TRP metabolites (e.g., kynurenine, quinolinic acid)	↑ ROS in endothelial cells ↓ cell viability ↓ NO signaling modulate immune-endothelial interactions via aryl hydrocarbon receptor activation [90,152,153]	Contribute to vascular damage and progression of both CKD and atherosclerosis. Proposed as a ‘missing link’ between renal failure, OS and premature CVD [153].

ADMA, asymmetric dimethylarginine; CVD, cardiovascular disease; CKD, chronic kidney disease; eGFR, estimated glomerular filtration rate; eNOS, endothelial nitric oxide synthase; ICAM-1, intercellular adhesion molecule 1; IS, indoxyl sulfate; LDL, low-density lipoprotein cholesterol; NADPH, nicotinamide adenine dinucleotide phosphate; NF-κB, nuclear factor kappa B; NO, nitric oxide; NOS, nitric oxide synthase; OS, oxidative stress; PCS, p-cresyl sulfate; Pi, inorganic phosphate; ROS, reactive oxygen species; SDMA, symmetric dimethylarginine; TRP, tryptophan metabolites; VCAM-1, vascular cell adhesion molecule 1; ↑, increase or upregulation; ↓, decrease or downregulation.

## Data Availability

No new data were created or analyzed in this study.

## Abbreviations

The following abbreviations are used in this manuscript:

CKD	chronic kidney disease
CAD	coronary artery disease
OS	oxidative stress
AGEs	advanced glycation end products
IL-6	interleukin-6
TNF-α	tumor necrosis factor alpha
NF-κB	nuclear factor kappa B
IL-1β	interleukin-1 beta
eNOS/NO	endothelial nitric oxide synthase/nitric oxide
IL-18	interleukin 18
CRP	C-reactive protein
hsCRP	high-sensitivity C-reactive protein
CKD-MBD	chronic kidney disease–mineral and bone disorder
FGF23	fibroblast growth factor 23
VSMC	vascular smooth muscle cells
ROS	reactive oxygen species
HD	hemodialysis
PD	peritoneal dialysis
SOD	superoxide dismutase
GSH	glutathione
MDA	malondialdehyde
GPx	glutathione peroxidase
MPO	myeloperoxidase
XO	xanthine oxidase
PGI_2_	prostacyclin
ADMA	asymmetric dimethylarginine
eGFR	estimated glomerular filtration rate
ICAM-1	intercellular adhesion molecule 1
VCAM-1	vascular cell adhesion molecule 1
FMD	flow-mediated dilation
miR-92a	microRNA-92a
RAGE	receptor for advanced glycation end products
IS	indoxyl sulfate
PCS	p-cresyl sulfate
TMAO	trimethylamine N-oxide
TG	triglycerides
VLDL	very-low-density lipoproteins
HDL	high-density lipoprotein
apoB-100	apolipoprotein B-100
ApoA-I	apolipoprotein A-I
oxLDL	oxidized low-density lipoprotein cholesterol
cLDL	carbamylated low-density lipoprotein cholesterol
coxLDL	carbamylated-oxidized low-density lipoprotein
SCFA	short-chain fatty acid
ZO-1	zonula occludens 1
LPS	lipopolysaccharide
TLR4	Toll-like receptor 4
sCD14	soluble CD14
CVD	cardiovascular disease
OCT	optical coherence tomography
VH-IVUS	virtual histology intravascular ultrasound
IB-IVUS	integrated backscatter intravascular ultrasound
CT	computed tomography
ACS	acute coronary syndrome
CMD	coronary microvascular dysfunction
CFR	coronary flow reserve
HFpEF	heart failure with preserved ejection fraction
